# Supplementary material for: ClarID: a human-readable and compact identifier specification for biomedical metadata integration
Source: J Biomed Semantics. 2026 Apr 24;17:9. doi: 10.1186/s13326-026-00349-6 (PMC13123180; doi:10.1186/s13326-026-00349-6)
Supplement: Supplementary file 1 — Supplementary Material 1: Additional file 1 (PDF): Implementation details [file 13326_2026_349_MOESM1_ESM.pdf]

Implementation details

Software

ClarID-Tools is implemented as a command-line interface (CLI) in Perl 5, chosen for its efficiency in handling structured text files and its simplicity for a reference implementation. The modular architecture and test suite ensure that reimplementation in other programming languages is straightforward, particularly with the support of modern development tools and large language models (LLM).

Internally, the tool uses *Moo* and *MooX::Options* modules to define required and optional parameters, apply validation logic, and enable context-sensitive behavior depending on entity type and encoding mode. For data handling, it integrates *YAML::XS* and *Text::CSV_XS* for high-performance parsing of codebooks and bulk tabular files, along with *JSON::Validator* for schema-based validation of the codebook structure. QR code generation is performed using the *qrencode* library for Linux.

During development, we explored fully externalizing the ClarID specification itself, i.e., the structural rules governing identifiers, such as field widths, regular expresions (regex) patterns, and transformations used for stub encoding (e.g., Base62). This was tested using JSON Schema. While attractive in theory, this approach quickly became impractical: managing the full specification through schema files led to greater complexity than the core implementation, with convoluted regex layers and transformations. Instead, we adopted a hybrid approach: some parameters (e.g., species, tissues, assays) remain configurable via the YAML codebook, while core structural rules are hardcoded for

simplicity. This approach maintains simplicity for implementers while keeping the specification transparent and reproducible across languages. The codebook itself is still validated against a JSON Schema to ensure consistency.

Although the current implementation focuses on identifiers for “subject” and “biosample” entities, the same principles (structured fields, consistent parsing rules, and YAML-based configuration) can be extended to additional entities such as cohorts, datasets, or experiments. As an interim solution (when code changes are not feasible), users may repurpose existing codebook fields to meet project-specific needs—for example, by using the “tissue” field to represent geographic location. While this departs from semantic accuracy, identifiers remain fully functional as long as the overall structure is preserved. Note that with minor code modifications, such extensions can be supported natively. Future updates or reimplementations, whether of the reference tool or by third parties, can reuse the same YAML-based configuration and structural logic to maintain full interoperability across environments.

Encoding and decoding logic (stub format)

The two formats (human-readable and stub) are fully interoperable and serve distinct purposes: the former enhances interpretability for human users, while the latter optimizes compactness for computational workflows. In the human-readable form, identifiers are generated by concatenating fields with hyphens, with the only transformation being the zero-padding of the numeric “subject_id” field. The stub form, by contrast, applies

compact transformations such as Base62 encoding for numeric fields, making it shorter but less directly interpretable. These transformations are described in detail below.

Encoding of ”project” (biosample) and “study” (subject)

Labels such as ‘TCGA_AML’ are arbitrary alphanumeric strings rather than simple numbers, so there is no generic way to compress them into a compact stub. If a shorter code is desired, users should define a corresponding alias in the YAML codebook; otherwise, ClarID will include the full label in both human-readable and stub formats.

Base62 transformation of “subject_id” (subject and biosample)

In ClarID, each subject’s numeric identifier (“subject_id”) is converted into a fixed-width alphanumeric stub using Base62 encoding with a 62-character alphabet (0–9, A–Z, a–z). By default, the stub width is three characters, configurable via the --*subject_id_base62_width*.

For “subject_id” = 0, the stub is simply “0” repeated to the chosen width. For values greater than zero, successive division and modulo operations by 62 are performed to extract characters. These are then left-padded with “0” to enforce the fixed width. For example, subject_id = 999 yields “G7,” which is padded to “0G7.”

Although Base62 encoding naturally produces variable-length strings, enforcing a fixed width ensures consistent parsing and supports up to 62^3 – 1 = 238,327 unique

individuals with a three-character stub. Larger cohorts can be accommodated by increasing the width using the --*subject_id_base62_width* flag.

Encoding of "condition" (subject and biosample)

The "condition" field is encoded by mapping each ICD-10 code to a numeric index using the reference ordering distributed with ClarID-Tools (icd10_order.json). That number is then converted to a fixed-width Base62 stub. The resulting alphanumeric code is fixed to a length of 3 characters to maintain compactness. This allows disease information to be stored concisely within the identifier, while still allowing for reverse mapping during decoding. This field supports multiple values: in the human-readable format they are separated by the '+' character, while in stub form they are concatenated using their stub codes.

Encoding of "species" (biosample)

Species "stub_code" is defined in the codebook. In the reference codebook, Base62 alphanumeric codes (0–9, A–Z, a–z) with a fixed width of two characters are used, supporting up to 3,844 species (with "00" code reserved for unknown values). Larger encoding spaces can be achieved by increasing the stub width (e.g., three characters support up to 238,328 values). ClarID-Tools does not enforce a specific encoding scheme for species; alternative encodings may be used if needed, provided the stub width remains consistent within a codebook. Optional taxonomy-based identifiers (tax_code, e.g., "MacMul" for *Macaca mulatta*) are included for reference but are not used in the stub format.

Encoding/decoding of "tissue", "sample_type" and "assay" (biosample)

These fields are expected to have a limited number of distinct values (dozens to hundreds), so instead of using dynamic base encoding, we adopted predefined stub_codes. This choice provides greater flexibility and interpretability while keeping stub strings short.

During decoding, the parser first handles fixed-width fields (e.g., subject_id) and then applies greedy reverse lookup for the variable-length fields. Stub codes are sorted in descending order of length to prevent prefix collisions. For example, with stub codes such as "PB" (peripheral blood), "T" (tumor), and "HI" (Hi-C sequencing), a stub like …PBTHI… will be correctly parsed into its respective metadata components. Although there is no strict length limit for stub codes, we recommend codes between 2 and 5 characters to maintain compactness and readability.

TSV/CSV data pre-processing script for ClarID-Tools

A custom Python 3 pre-processing script (included in the ClarID-Tools repository) reads raw TSV/CSV data, applies YAML-defined field-by-field transformations (e.g., trimming, value mapping, multi-value normalization, etc.), producing a standardized CSV ready for ClarID-Tools.

Pre-processing of timepoint's "duration" (biosample)

To keep the component to 3 characters (human format) or 2 (stub format), the pre-processing script converts a numeric day count into the smallest allowed ISO-8601 bin

(P[0-9][DWMY]) that can represent it without exceeding 9 in that unit, escalating from days → weeks → months → years, with rounding as configured.

Codebook (v0.03)

The latest release of the codebook is available at the project repository. The full codebook is too lengthy to include here; an example is provided.

```
metadata:
  version: "0.03"
  local_version: "CNAG-GDC-v1" # optional
  author: "Manuel Rueda"
  center: "CNAG"
  date: "2026-04-01"
  description: "CNAG's ClarID codebook."
  repository: "https://github.com/CNAG-Biomedical-Informatics/clarid-tools"

entities:
  biosample:
    project:
      TCGA-AML:
        code: TCGA_AML
        stub_code: AML
        label: "TCGA Acute Myeloid Leukemia"
        id: "NCIT:C17998"
    species:
      Human:
        code: HomSap
        stub_code: "01"
        label: "Homo sapiens"
        id: "NCBITaxon:9606"
        tax_code: MPH
    tissue:
      Liver:
        code: LIV
        stub_code: L
        label: "Liver"
        id: "UBERON:0002107"
    sample_type:
      Tumor:
        code: TUM
        stub_code: T
        label: "Neoplasm"
        id: "NCIT:C3262"
    assay:
      RNA_seq:
        code: RNA
        stub_code: R
```

```
        label: "RNA-seq"
        id: "EFO:0008896"
    timepoint:
      Baseline:
        code: BSL
        stub_code: "B"
        label: "Baseline"
        id: "NCIT:C25213"
    condition_pattern:
      regex: "^([A-Z]\\d{2}(?:\\.\\d+)?)$"
      code_format: "%s"
      stub_format: "%s"
    duration_pattern:
          regex: '^(?:P?(\d+)([DWMY])|P?(0)(N))$'
      code_format: "P%d%s"
      stub_format: "%d%s"
    batch_pattern:
      regex: "^(\\d{1,2})$"
      code_format: "B%02d"
      stub_format: "%02d"
    replicate_pattern:
      regex: "^(\\d{1,2})$"
      code_format: "R%02d"
      stub_format: "%02d"

  subject:
    study:
      TCGA-AML:
        code: TCGA_AML
        stub_code: AML
        label: "TCGA Acute Myeloid Leukemia"
        id: "NCIT:C17998"
    type:
      Case:
        code: Case
        stub_code: C
        label: "Case Study"
        id: "NCIT:C15362"
    sex:
      Male:
        code: Male
        stub_code: M
        label: "Male"
        id: "PATO:0000384"
    age_group:
      Age20to29:
        code: A20_29
        stub_code: A2
        label: "Age 20-29"
        id: "NCIT:C20192"
```

Specification (v0.03)

The codebook and the ClarID-Tools specification are versioned synchronously. The latest releases, example codebooks, and version history are openly maintained in the ClarID-Tools repository to support reuse and community contributions. For the most recent specification, please refer to this link.

Biosample - Human format

Delimiter: -

#	Component	Source field	Type	Pattern / Format	Built from
1	Project	project.code	string	free string	codebook value (if present)
2	Species	species.code	string	6-letter binomial acronym (e.g. HomSap)	codebook value
3	Subject ID	subject_id	integer -> str	zero-pad to 5 digits (default)	sprintf("%0${pad}d", $sid)
4	Tissue	tissue.code	string	exactly 3 letters [A-Z]{3}	codebook value
5	Sample Type	sample_type.code	string	exactly 3 letters [A-Z]{3}	codebook value
6	Assay	assay.code	string	exactly 3 letters [A-Z]{3}	codebook value
7	Condition	condition	string	ICD-10 diagnose code(s) [A-Z]\d{2}(?:\.\d+)?(<=10)	used verbatim (or concatenated with +)
8	Timepoint	timepoint	string	alphanumeric events e.g. Baseline	codebook value
9	Duration	duration	string	ISO 8601 3-char (P1D,P7W,P3M,P1Y) or P0N (Not Available)	duration_pattern
10	Batch (opt)	batch	integer -> str	B%02d (e.g. B01)	batch_pattern

11	Replicate (opt)	replicate	integer -> str	R%02d (e.g. R05)	replicate_pattern

Biosample - Stub format

Delimiter: (none)

#	Component	Source field	Type	Pattern / Format	Built from
1	Project stub	project.stub_code	string	free string	codebook value
2	Species stub	species.stub_code	string	codebook-defined stub width (Base62 alphabet recommended)	codebook value
3	Subject stub	subject_id	integer -> Base62	width 3 (default) - max 238,327	3-char Base62 from integer
4	Tissue stub	tissue.stub_code	string	1-3 chars	codebook value
5	Sample Type stub	sample_type.stub_code	string	1-3 chars	codebook value
6	Assay stub	assay.stub_code	string	1-3 chars	codebook value
7	Condition stub	condition	code -> Base62	N x 3-char Base62 stubs + 2-digit count (%02d)	codebook order + 3-char Base62 from integer
8	Timepoint stub	timepoint.stub_code	string	1-2 chars	codebook value
9	Duration stub	duration	string	digits+unit (e.g. 7W)	duration_pattern
10	Batch stub (opt)	batch	integer	B%02d (e.g. B01)	batch_pattern
11	Replicate stub (opt)	replicate	integer	R%02d (e.g. R05)	replicate_pattern

Subject - Human format

Delimiter: -

#	Component	Source field	Type	Pattern / Format	Built from
1	Study	study	string	free string	codebook value (if present)
2	Subject ID	subject_id	integer -> str	zero-pad to 5 digits (default)	sprintf("%0${pad}d",$sid)
3	Type	type.code	string	codebook codes	codebook value
4	Condition	condition	string	ICD-10 diagnose code(s): [A-Z]\d{2}(?:\.\d+)? (<=10)	used verbatim (or concatenated +)
5	Sex	sex.code	string	codebook codes	codebook value
6	Age Group	age_group.code	string	codebook codes	codebook value

Subject - Stub format

Delimiter: (none)

#	Component	Source field	Type	Pattern / Format	Built from
1	Study stub	study.stub_code	string	free string	codebook value (if present)
2	Subject ID stub	subject_id	integer -> Base62	width 3 (default) - max 238,327	3-char Base62 from integer
3	Type stub	type.stub_code	string	1 char	codebook value
4	Condition stub	condition	code -> Base62	N x 3-char Base62 stubs + 2-digit count (%02d)	codebook order + 3-char Base62 from integer
5	Sex stub	sex.stub_code	string	1 char	codebook

					value
6	Age Group stub	age_group.stub_code	string	2 chars	codebook value
