# Supplementary material for: ClarID: a human-readable and compact identifier specification for biomedical metadata integration
Source: J Biomed Semantics. 2026 Apr 24;17:9. doi: 10.1186/s13326-026-00349-6 (PMC13123180; doi:10.1186/s13326-026-00349-6)
Supplement: Supplementary file 2 — Supplementary Material 2: Additional file 2 (PDF): Supplementary table. Table ST1: Implementation challenges and solutions [file 13326_2026_349_MOESM2_ESM.pdf]

Supporting Table 1: Implementation challenges and solutions

Data pre-processing	
**Faced challenges**	**Solution**
CSV is flexible but lacks enforced structure; missing values and inconsistent codes are common	We developed a preprocessing script, guided by a configuration file, to standardize raw CSV rows and columns. The script handles missing entries, harmonizes inconsistent codes, and produces a clean CSV suitable for bulk processing with ClarID-Tools. The scripts are available at: https://github.com/CNAG-Biomedical-Informatics/clarid-tools/tree/main/utils/csv/
**ClarID-Tools (CLI and module)**	
**Faced challenges**	**Solution**
Standardization of components' vocabulary	In the codebook, ontology terms were used to define properties where possible (e.g., tissues with UBERON). For diseases and technical fields like batch/replicate, regex rules were applied. The structure remains flexible, allowing users to extend it as needed.
Externalized schema vs. hard-coded specification	Full JSON Schema externalization proved impractical; we adopted a hybrid approach with YAML-configurable parameters and some hardcoded elements, while validating the codebook itself against a JSON Schema.
Compaction of human → stub formats	Certain fields required Base62 encoding to condense information. The field width was restricted for parsing but can be adjusted through CLI parameters.
