# Supplementary material for: ClarID: a human-readable and compact identifier specification for biomedical metadata integration
Source: J Biomed Semantics. 2026 Apr 24;17:9. doi: 10.1186/s13326-026-00349-6 (PMC13123180; doi:10.1186/s13326-026-00349-6)
Supplement: Supplementary file 3 — Supplementary Material 3: Additional file 3 (PDF): Supplementary figures. Figure SF1: Example QR codes of ClarID identifiers [file 13326_2026_349_MOESM3_ESM.pdf]

Supporting Figure 1

(a)

AsthmaCohort-01002-Control-J98.51-Female-A50_59

AsthmaCohort-01004-Case-J98.51-Male-A40_49

COPDStudy-01001-Case-J98.51-Male-A40_49

COPDStudy-01003-Control-J98.51-Female-A50_59

(b)

CNAG_Test-DanRer-00003-BLO-NOR-WES-I46-SUR-P1M-B03-R01

CNAG_Test-HomSap-00001-LIV-NOR-RNA-C22.0-BSL-P0D-B01-R05

CNAG_Test-MusMus-00002-BRN-TUM-CHI-C71.0-TRT-P7W-B02-R02

CNAG_Test-RatNor-00004-KID-NOR-LCMS-C66-CHL-P3Y-B01-R10

Figure SF1. Example QR (Quick Response) codes with their decoded ClarID identifiers shown alongside each image, for (a) a "subject" entity and (b) a "biosample" entity. The QR codes can be read by any QR-enabled device, such as a smartphone.
